# Supplementary material for: Immigrants resettlement in developing countries: A data-driven decision tool applied to the case of Venezuelan immigrants in Colombia
Source: PLoS One. 2022 Jan 25;17(1):e0262781. doi: 10.1371/journal.pone.0262781 (PMC8789124; doi:10.1371/journal.pone.0262781)
Supplement: S1 Appendix — (DOCX) [file pone.0262781.s001.docx]

1. Activities auxiliary to financial intermediation
2. Mining and coal extraction
3. Insurance and pension plan financing
4. Water collection, purification and distribution
5. Manufacture of textile products
6. Activities complementary/auxiliary to transportation
7. Manufacture of radio and television equipment
8. Private households with domestic service
9. Wholesale and retail trade
10. Manufacture of chemical substances and products
11. Cultural and sporting activities
12. Manufacture of medical instruments
13. Hotels, restaurants, bars and the like
14. Construction
15. Manufacture of motor vehicles
16. Activities of not previously classified associations
17. Manufacture of electrical machinery and apparatus
18. Computer and related activities
19. Postal and telecommunications activities
20. Collection, treatment and disposal of waste
21. Publishing/recording activities
22. Manufacture of machinery and equipment not previously classified
23. Installation, maintenance and repair of machinery
24. Tanning and dressing of hides and skins, leather working
25. Sanitation and waste disposal
26. Fishing service activities
27. Manufacture of furniture
28. Financial intermediation
29. Education
30. Health and social work services
31. Real estate activities
32. Manufacture of other transport equipment
33. Offshore organizations
34. Food processing
35. Forestry and logging
36. Professional, scientific and technical activities
37. Manufacture of wearing
38. Other service activities
39. Manufacture of fabricated metal products
40. Electricity, gas and steam supply
41. Public administration and defense
42. Manufacture of paper and cardboard products
43. Other business activities
44. Manufacture of tobacco products
45. Maritime transportation
46. Agriculture, livestock and hunting
47. Manufacture of rubber and plastic products
48. Other manufacturing
49. Manufacture of basic metal products
50. Air transport
51. Renting of machinery and equipment without operators
52. Manufacture of wood products
53. Recycling of metallic and non-metallic materials
54. Manufacture of non-metallic mineral products
55. Land transport
