# Supplementary material for: Immigrants resettlement in developing countries: A data-driven decision tool applied to the case of Venezuelan immigrants in Colombia
Source: PLoS One. 2022 Jan 25;17(1):e0262781. doi: 10.1371/journal.pone.0262781 (PMC8789124; doi:10.1371/journal.pone.0262781)
Supplement: S1 Table — (DOCX) [file pone.0262781.s002.docx]

| **Location** | Respondent |
| --- | --- |
| NORTE DE SANTANDER | 2583 |
| ATLANTICO | 2290 |
| GUAJIRA | 2042 |
| MAGDALENA | 1936 |
| CESAR | 1561 |
| SANTANDER | 1239 |
| ANTIOQUIA | 1200 |
| BOGOTA | 1161 |
| BOLIVAR | 1072 |
| VALLE | 992 |
| SUCRE | 944 |
| RISARALDA | 582 |
| QUINDIO | 551 |
| META | 537 |
| CORDOBA | 399 |
| BOYACA | 357 |
| CALDAS | 328 |
| CAUCA | 293 |
| TOLIMA | 260 |
| NARIÑO | 213 |
| HUILA | 198 |
| CUNDINAMARCA | 182 |
| CHOCO | 114 |
| CAQUETA | 98 |
