# Supplementary material for: Immigrants resettlement in developing countries: A data-driven decision tool applied to the case of Venezuelan immigrants in Colombia
Source: PLoS One. 2022 Jan 25;17(1):e0262781. doi: 10.1371/journal.pone.0262781 (PMC8789124; doi:10.1371/journal.pone.0262781)
Supplement: S2 Table — (DOCX) [file pone.0262781.s003.docx]

| **Type of indicator** | **Title** | **Definition** |
| --- | --- | --- |
| **Development** | Human Development Index (HDI) | The HDI assesses long-term progress in 3 basic dimensions: (1) long and healthy life, (2) access to education, and (3) decent standard of living. Higher HDI values indicate a better standard of living. |
|  | Multidimensional Poverty Index (IPM) | The IPM takes into account 5 dimensions measured with 15 indicators and is based on the deprivations that individuals have in relation to the relevant conditions that guarantee a decent standard of living, such as education, employment, health, housing and access to public services. |
| **Economy** | Monetary poverty and extreme poverty | It measures the percentage of people living with incomes below a specific threshold. |
|  | Gross Domestic Product (GDP) | GDP gives the monetary value of the production of final goods and services per capita. |
| **Employability** | Unemployment rate | Percentage of the working population that is unemployed. |
|  | Ratio between job offers and demand | It is the ratio of total job openings to vacancies published per year in each location . |
|  | Child labor | Proportion of children aged 12 to 17 in the household who are out of the labor market. |
|  | Long-term unemployment rate | Proportion of the economically active household population that is unemployed in the last 12 months. |
|  | Rate of informal employment | Percentage of the labor force informally employed. |
| **Health** | Health coverage | Coverage is given by the percentage of people affiliated to health services. |
|  | Barriers to access to health services | proportion of persons in households with an unmet health need due to lack of access to health services. |
|  | Barriers to early child care services | Proportion of children 0 to 5 years old without simultaneous access to health and nutrition. |
|  | Subsidized insurance scheme | The subsidized regime covers people who cannot afford to pay for their affiliation to health services. |
|  | Contributory insurance scheme | The contributory regime is the system that covers workers who contribute a portion of their salary to have health coverage for themselves and their families. |
|  | Uninsured population | Percentage of people without health insurance. |
| **Education** | Illiteracy | Percentage of people 15 years of age and older who cannot read and write |
|  | Low level of education | The low education rate is measured by the percentage of people 15 years and older who have attained less than 9 years of education. |
|  | School absenteeism rate | Proportion of children and youth between the ages 7 and 17 who do not attend school. |
| **Well-being** | Housing deficit | Refers to households living in private dwellings that present housing shortages due to quantitative and qualitative deficits and therefore require new housing or an improvement or expansion of the housing unit in which they live. |
|  | Critical overcrowding rate | Number of persons per bedroom. Cutoff point: 3 persons. |
|  | No access to improved water sources | Households that obtain water for food preparation from wells, rivers, springs, or other sources. |
