# Supplementary material for: Immigrants resettlement in developing countries: A data-driven decision tool applied to the case of Venezuelan immigrants in Colombia
Source: PLoS One. 2022 Jan 25;17(1):e0262781. doi: 10.1371/journal.pone.0262781 (PMC8789124; doi:10.1371/journal.pone.0262781)
Supplement: S3 Table — (DOCX) [file pone.0262781.s004.docx]

|  | **Individual probabilities** | | | **Mapped probabilities** | | |
| --- | --- | --- | --- | --- | --- | --- |
| **Location** | **Minimum** | **Maximum** | **Mean** | **Minimum** | **Maximum** | **Mean** |
| Antioquia | 0,015 | 0,857 | 0,268 | 0,018 | 0,985 | 0,383 |
| Atlantico | 0,008 | 0,840 | 0,247 | 0,009 | 0,975 | 0,357 |
| Bogota | 0,010 | 0,841 | 0,256 | 0,010 | 0,978 | 0,368 |
| Bolivar | 0,007 | 0,816 | 0,224 | 0,007 | 0,937 | 0,325 |
| Boyaca | 0,007 | 0,817 | 0,229 | 0,007 | 0,935 | 0,332 |
| Caldas | 0,008 | 0,803 | 0,240 | 0,009 | 0,938 | 0,347 |
| Caqueta | 0,009 | 0,802 | 0,233 | 0,009 | 0,929 | 0,337 |
| Cauca | 0,007 | 0,793 | 0,208 | 0,009 | 0,927 | 0,303 |
| Cesar | 0,005 | 0,777 | 0,182 | 0,005 | 0,943 | 0,267 |
| Cordoba | 0,005 | 0,778 | 0,183 | 0,005 | 0,943 | 0,269 |
| Cundinamarca | 0,004 | 0,783 | 0,183 | 0,004 | 0,944 | 0,270 |
| Choco | 0,005 | 0,782 | 0,179 | 0,005 | 0,943 | 0,263 |
| Huila | 0,005 | 0,787 | 0,170 | 0,005 | 0,945 | 0,252 |
| La Guajira | 0,004 | 0,784 | 0,158 | 0,004 | 0,943 | 0,234 |
| Magdalena | 0,004 | 0,776 | 0,155 | 0,004 | 0,937 | 0,230 |
| Meta | 0,004 | 0,774 | 0,160 | 0,004 | 0,937 | 0,239 |
| Narino | 0,004 | 0,770 | 0,159 | 0,004 | 0,936 | 0,237 |
| Norte de Santander | 0,004 | 0,771 | 0,149 | 0,004 | 0,924 | 0,222 |
| Quindio | 0,005 | 0,769 | 0,181 | 0,006 | 0,919 | 0,267 |
| Risaralda | 0,006 | 0,805 | 0,196 | 0,007 | 0,903 | 0,288 |
| Santander | 0,005 | 0,793 | 0,191 | 0,005 | 0,888 | 0,282 |
| Sucre | 0,006 | 0,778 | 0,193 | 0,006 | 0,886 | 0,283 |
| Tolima | 0,007 | 0,761 | 0,218 | 0,007 | 0,901 | 0,317 |
| Valle | 0,021 | 0,756 | 0,283 | 0,021 | 0,939 | 0,401 |
