# Supplementary material for: Immigrants resettlement in developing countries: A data-driven decision tool applied to the case of Venezuelan immigrants in Colombia
Source: PLoS One. 2022 Jan 25;17(1):e0262781. doi: 10.1371/journal.pone.0262781 (PMC8789124; doi:10.1371/journal.pone.0262781)
Supplement: Attachment — Submitted filename: Editor comment on PONE-D-21-24079.docx [file pone.0262781.s005.docx]

Comments

The selection of research topic entitled (**Manuscript Number PONE-D-21-24079) “Immigrants resettlement in developing countries: a data-driven decision tool applied to the case of Venezuelan immigrants in Colombia”** is very advanced , and it is more applicable to existing scenarios .The author has been attempted to describe the entire research intervention of	a data driven support model for immigrants . During the process of reviewing , I have found that , there is	merit in the research paper and I have been accepted for publication by cross checking and addition of newer bibliography.

**Dr Basavarajaiah DM**

**Editor –Academic**
